# TRANSGENDER OLDER ADULTS HOSPITAL USE IN SOUTH CAROLINA

**DOI:** 10.1093/geroni/igae098.2488

**Published:** 2024-12-31

**Authors:** Jennifer May, Hanna Smyles, Swann Adams

**Affiliations:** University of South Carolina, Columbia, South Carolina, United States; University of South Carolina, Columbia, South Carolina, United States; University of South Carolina, Columbia, South Carolina, United States

## Abstract

Transgender adults in the South represent a unique population with health disparities and care inequities due to longstanding false stereotypes and discrimination surrounding their transgender status. Transgender adults report less access to health insurance, lower quality of life, and severe mental distress compared to the general U.S. population. There is a paucity of literature identifying the hospital patterns of transgender older adults (≥50 years). This study examines hospital utilization among transgender older adults (≥50 years) in South Carolina to describe their demographic characteristics and use of inpatient, outpatient surgery, and emergency department services. We conducted a secondary analysis of restricted hospital claims data from 2000 to 2022; identified transgender adults, using ICD-9 and ICD-10 codes, who used hospital services in South Carolina. Patient demographics and clinical data were analyzed using descriptive statistics. We identified 382 unique individual transgender adults with our algorithm. Most identified as White (76%); 70% were in the age range of 50-59; sex as reported on the administrative record was mostly male at 51%. 78% (n=299) of transgender adults were from urban areas. Transgender adults could have had more than one type of hospital use with 272 unique individuals using inpatient hospital services, 334 unique individuals using outpatient hospital services, and 346 unique individuals using emergency room services. Understanding the hospital use and the urban vs rural location of the transgender adult population can help identify state resource allocation, capacity building, and creating a foundation for bolstering health services and reducing health inequities.

